# Population genetics analysis during the elimination process of *Plasmodium falciparum* in Djibouti

**DOI:** 10.1186/1475-2875-12-201

**Published:** 2013-06-13

**Authors:** Bouh Abdi Khaireh, Ashenafi Assefa, Hawa Hassan Guessod, Leonardo K Basco, Mohamed Abdi Khaireh, Aurélie Pascual, Sébastien Briolant, Samatar Mohamed Bouh, Ismaïl Hassan Farah, Habib Moussa Ali, Abdoul-Ilah Ahmed Abdi, Mouna Osman Aden, Zamzam Abdillahi, Souleiman Nour Ayeh, Houssein Youssouf Darar, Jean-Louis Koeck, Christophe Rogier, Bruno Pradines, Hervé Bogreau

**Affiliations:** 1 Unité de Parasitologie, Département d’Infectiologie de Terrain, Institut de Recherche Biomédicale des Armées, Allée du Médecin Colonel E. Jamot, Parc du Pharo, BP 60109, 13262, Marseille Cedex 07, France; 2 Aix Marseille Université, Unité de Recherche sur les Maladies Infectieuses et Tropicales Emergentes, UM 63, CNRS 7278, IRD 198, INSERM 1095, Marseille, France; 3 Service de Santé des Forces Armées Djiboutiennes, DSS/RQG, BP46, Djibouti, Republic of Djibouti; 4 Institut de Recherche Médicinale, Centre d’Etude et de Recherche de Djibouti, BP 486, Route de l’Aéroport, Republic of Djibouti; 5 Ethiopian Health and Nutrition Research Institute (EHNRI), Addis Ababa, Ethiopia; 6 Programme National de Lutte contre le VIH/SIDA, la Tuberculose et le Paludisme, BP 1157, Ministère de la Santé, Republic of Djibouti; 7 Ministère de la Santé, Institut National de Santé Publique, BP 1157, Djibouti, Republic of Djibouti; 8 Service des Maladies Infectieuses et Tropicales, Hôpital Général Peltier, Djibouti, Republic of Djibouti; 9 Centre Hospitalier des Armées Bouffard, Djibouti, Republic of Djibouti; 10 Institut Pasteur de Madagascar, 101 Antananarivo, Madagascar

**Keywords:** Microsatellites, Molecular epidemiology, Genetic diversity, Drug resistance, Malaria elimination, Pyrimethamine, Horn of Africa

## Abstract

**Background:**

Case management of imported malaria within the context of malaria pre-elimination is increasingly considered to be relevant because of the risk of resurgence. The assessment of malaria importation would provide key data i) to select countries with propitious conditions for pre-elimination phase and ii) to predict its feasibility. Recently, a sero-prevalence study in Djibouti indicated low malaria prevalence, which is propitious for the implementation of pre-elimination, but data on the extent of malaria importation remain unknown.

**Methods:**

Djiboutian plasmodial populations were analysed over an eleven-year period (1998, 1999, 2002 and 2009). The risk of malaria importation was indirectly assessed by using plasmodial population parameters. Based on 5 microsatellite markers, expected heterozygosity (H.e.), multiplicity of infection, pairwise Fst index, multiple correspondence analysis and individual genetic relationship were determined. The prevalence of single nucleotide polymorphisms associated with pyrimethamine resistance was also determined.

**Results:**

Data indicated a significant decline in genetic diversity (0.51, 0.59, 0.51 and 0 in 1998, 1999, 2002 and 2009, respectively) over the study period, which is inconsistent with the level of malaria importation described in a previous study. This suggested that Djiboutian malaria situation may have benefited from the decline of malaria prevalence that occurred in neighbouring countries, in particular in Ethiopia. The high Fst indices derived from plasmodial populations from one study period to another (0.12 between 1999 and 2002, and 0.43 between 2002 and 2009) suggested a random sampling of parasites, probably imported from neighbouring countries, leading to oligo-clonal expansion of few different strains during each transmission season. Nevertheless, similar genotypes observed during the study period suggested recurrent migrations and imported malaria.

**Conclusion:**

In the present study, the extent of genetic diversity was used to assess the risk of malaria importation in the low malaria transmission setting of Djibouti. The molecular approach highlights i) the evolution of Djiboutian plasmodial population profiles that are consistent and compatible with Djiboutian pre-elimination goals and ii) the necessity to implement the monitoring of plasmodial populations and interventions at the regional scale in the Horn of Africa to ensure higher efficiency of malaria control and elimination.

## Background

According to the World Malaria Report 2012 [1], there were about 219 million cases of malaria (with an uncertainty range [10 to 90 percentile] of 154 million to 289 million) and an estimated 660,000 deaths in 2010 (with an uncertainty range of 610,000 to 971,000). Malaria mortality rates have fallen by more than 25% globally since 2000 and by 33% in the World Health Organization (WHO) African region [2]. Today, the WHO is considering that in some regions characterized by favourable conditions, malaria elimination has become a realistic goal [3-5]. As a first step, these areas are eligible for pre-elimination stage. Local malaria transmission can subsequently be interrupted in these areas through incremental stages. A progressive decline in malaria transmission is expected to reduce the surface area of malaria-endemic region, country by country, from hypo-endemic to hyper-endemic areas [6].

However, malaria control and the pre-elimination process may be hampered by multiple insecticide and drug resistance of mosquito vectors and malaria parasites, respectively. In order to limit the impact of drug-resistant malaria and prevent its spread, monitoring of drug resistance is strongly recommended [5]. Moreover, imported malaria may hamper malaria control and cause malaria resurgence in countries that have reached the elimination phase [7,8]. The higher the numbers of imported cases in a country in the pre-elimination phase, the greater will be the risk that malaria returns and even rebounds, and more resources for combating malaria will be required to attain elimination [9]. Therefore, according to the incremental elimination strategy, areas with limited cases of imported malaria should be enlisted first in the pre-elimination phase. When elimination is attained in those areas, malaria elimination in neighbouring areas with higher malaria transmission level may be considered.

Since the level of local malaria endemicity and the number of imported cases are two key factors for the assessment of feasibility and estimation of resources required for the pre-elimination step, national surveillance system in a country aiming for pre-elimination should focus on these two determinants. To that end, classical sero-epidemiological methods as described by Corran *et al*. [10] and Drakeley *et al*. [11] may be completed by a population genetics approach which could take advantage of the population plasticity driven by evolutionary forces. For example, selection pressure or migrations alter parasite populations, and these modifications in plasmodial population would lead to genetic changes, which can be monitored. Analysis of allelic frequencies in plasmodial populations is an efficient way to describe such micro-evolution [12-20].

As allelic frequencies within parasite populations are summarized by genetic diversity defined by expected heterozygosity (He), [*H*_*e*_ = [*n*/(*n* − 1)][1 − *Σ*^n^_i = 1_*p*^2^_*i*_] [21], the determination of He is an efficient way to monitor changes occurring at the population level. Interestingly, He is positively correlated with local malaria transmission level [22-26]. Low local transmission levels are associated with low He [27,28]. Conversely, high transmission levels are associated with an increase in He [24,29]. He values may also be associated with human migrations which appear to be the main parasite ‘transporter and provider’ and therefore a ‘promoter’ of genetic diversity [22,30,31], even over long distances [32]. This is consistent with the correlation observed between human migration rate and genetic similarities among malaria parasite populations in an archipelago model [33]. Local levels of malaria transmission and the number of imported malaria cases are therefore positively correlated with genetic diversity. Since pre-elimination programme aims to decrease local transmission level and control parasite importations, both of these factors together should be associated with a decrease in plasmodial genetic diversity (He) during the process of pre-elimination.

Additional information on the evolution of allelic frequencies may be obtained from Fst index [34]. This index may be considered as a measure of the variance in allelic frequencies between populations [35]. Pairwise comparison between years over a study period highlights the global evolution and allows better understanding of the parasite turnover rate (i.e. high pairwise Fst between years is consistent with high turnover). Analysis of genotype frequencies and their similarity is also informative to assess and determine the origin of imported malaria cases. It may be possible to identify genetic lineage or recurrent genotypes. In order to derive valid data from genetic analysis, an establishment of genotype database from eligible areas is necessary before the pre-elimination process. In this way, it will be possible to monitor the impact of human actions on *Plasmodium falciparum* populations.

The Republic of Djibouti exhibits an interesting malaria situation to illustrate the usefulness of population genetics analysis within the context of malaria elimination. This country should be eligible for pre-elimination [6,36,37]. Djibouti (geographic coordinates of Djibouti city, the capital of the country, 11°36’N 43°10’E) [38] was formerly known to be a meso- to hypo-endemic country with unstable malaria [39-41]. *P*. *falciparum* and *P*. *vivax* co-exist in the country, but the former is the predominant (> 80%) species. The major vector is *Anopheles arabiensis*[1,42,43]. Its climate is semi-arid, with a mean annual rainfall of 147 mm [38]. Over the past 12 years, malaria incidence, as well as recurrence of epidemics, has decreased [44]. The epidemiologic strata have been illustrated by few micro-epidemics [32], with the exception of a major malaria outbreak that occurred in 1999 [45]. Sero-prevalence data from 2002 [43] confirmed the existence of residual malaria foci. In the city of Djibouti, hotspots occurred on both sides of the main wadi (i.e., Ambouli wadi) and in the quarters of Arhiba and Balbala 4 (Additional file 1 from Khaireh *et al*. [43]) where large populations of migrants who travel regularly to and/or from Ethiopia reside [46]. Across the Republic, *P*. *falciparum* sero-prevalence rates were higher in the southern regions (Dikhil and Ali-Sabieh), particularly along the land routes to Ethiopia, i.e., Tammiro/As-Eyla and Ali-Sabieh (Additional file 2 from Khaireh *et al*. [43]). These roads are used frequently by professional truckers, private users, and migrants because they are the only two roads connecting Djibouti and Ethiopia [47].

Recently, a national sero-prevalence investigation including 7,151 individuals (from November 2008 to January 2009) throughout the country (150 clusters of 75 households from the capital [40%] and districts [60%]) confirmed the very low malaria prevalence (0.58%) [48]. National sero-prevalence data are consistent with the decrease in malaria incidence monitored by three main medical services in the city of Djibouti (Additional file 3) where almost two-thirds of the population reside (61.7%, according to the official Djiboutian publication of census [49]). Considering this high Djiboutian urbanization and movement of people between the capital and the rest of the country [49], this correspondence between inside and outside the capital was expected. After the outbreak in 1999, the first decrease in malaria incidence occurred in 2001 (4-fold decrease) and the second decrease in 2006 (11-fold decrease) (Additional file 3). Although the decrease in malaria burden was in favour of the Djiboutian eligibility for pre-elimination step, there are no field data on the risk of malaria importation that could hamper the elimination process. In order to adapt malaria control programme in this country, it would be useful to characterize the circulating *P*. *falciparum* isolates in Djibouti and importations from neighbouring countries.

In the late 1980s, a study showed multiple events of malaria importation from neighbouring countries [41]. Moreover, previous molecular data suggested the existence of parasite exchange sufficient to provide a moderate genetic diversity [32,45], despite seasonal interruption of local malaria transmission [39,45]. However, the actual extent of imported malaria remains unknown. What is the genetic diversity of imported malaria? What is the parasite turnover rate? The success of the pre-elimination programme will depend, in part, on the responses to these questions.

To complete the understanding of Djiboutian malaria epidemiologic strata within the context of malaria pre-elimination, an indirect genetic approach was adopted. Analyses of genetic diversity, as well as those of relationships among parasites collected over a period of 11 years, shed light on the above-mentioned questions. Multilocus microsatellite genotyping was performed in *P*. *falciparum* isolates collected over 11 years (1998, 1999, 2002, and 2009). Based on WHO recommendations on drug resistance monitoring [3,5] and the fact that sulphadoxine-pyrimethamine is the current partner molecule of artesunate (artemisinin-based combination therapy [ACT]) for the first-line treatment employed in Djibouti since 2008 [50], polymorphisms associated with resistance to pyrimethamine were also genotyped.

## Methods

### *Plasmodium falciparum* isolates

**Figure 1:**
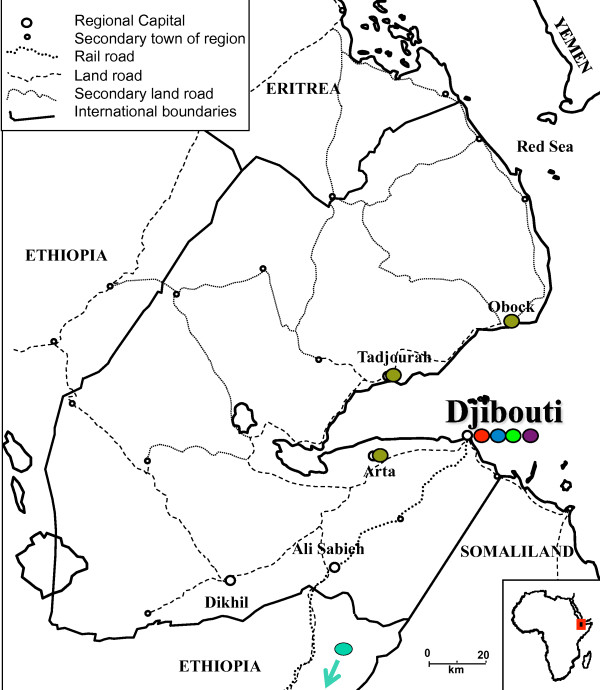
**Locations of *Plasmodium falciparum*-positive cases sampled in the Republic of Djibouti (1998, 1999, 2002, and 2009) Sampling sites where *Plasmodium falciparum *cases were diagnosed with rapid diagnostic tests and confirmed by PCR.** Forty-six blood samples were collected from September to December 1998 (red dot), 61 in April 1999 (blue dot), and 32 from March to May 2002 (light green dot) from symptomatic patients with *P*. *falciparum* who had not travelled outside the city of Djibouti during the preceding month and declared not to have taken any antimalarial drug before blood sampling. In 2009, 42 Djiboutian samples were collected from November 2008 to January 2009 by the Djiboutian Malaria Control Unit (Ministry of Health) (khaki green) [48]. One blood sample was obtained from a Djiboutian military recruit who presented with clinical malaria (purple dot) in January 2009 and who stayed at the training camp in Debrezeit (Ethiopia) for 10 months prior to blood collection. Three blood samples provided by the Department of Parasitology and Vector Borne Diseases (Ethiopian Health and Nutrition Research Institute) were collected in Southeast Ethiopia (dark green dot) in 2008. According to the official census of Djiboutian population, Djibouti exhibits a high urbanization rate. Djibouti city, Ali Sabieh, Dikhil, Tadjourah, Arta, and Obock account for 62%, 11%, 10%, 9%, 5%, and 4% of the national population, respectively [49].

Isolates were collected four times over an 11-year period (1998, 1999, 2002, and 2009). The first three investigations were conducted at the Centre Hospitalier des Armées Bouffard, a French military hospital in Djibouti city serving Djiboutian military and native civilians residing in the city, and other public health facilities in Djibouti city. Blood samples were collected from symptomatic patients with *P*. *falciparum* who had not travelled outside the city of Djibouti during the preceding month and who denied self-medication with an anti-malarial drug before consultation. Forty-six blood samples were collected between September and December 1998, 61 in April 1999, and 32 between March and May 2002. Venous blood (5 mL) was collected in ethylene diamine tetra acetic acid (EDTA)-coated Vacutainer® tubes (Becton Dickinson, Rutherford, NJ, USA). Aliquots of freshly collected blood were kept at −20°C until DNA extraction. In 2009, during the fourth investigation, 42 samples were provided by the Djiboutian Malaria Control Unit (Ministry of Health). These samples, collected during an investigation of malaria seroprevalence detailed elsewhere, were obtained from three sites outside Djibouti city: Arta, Tadjourah, and Obock [48] (Figure 1). One blood sample was obtained from a Djiboutian military recruit who presented with clinical malaria in January 2009 after staying at the military training camp in Debrezeit (Ethiopia) for 10 months prior to blood collection. Three additional isolates from Ethiopia sampled in 2008 were provided by the Department of Parasitology and Vector Borne Diseases (Ethiopian Health and Nutrition Research Institute).

Blood samples were spotted onto Whatman® 3 MM filter paper, and all samples were frozen and kept at −20°C in the national laboratory at Hôpital General Peltier in Djibouti city. The study was cleared and approved by the Djiboutian Ministry of Health (authorization no. 48/INSP/MS/2012). Informed consent was obtained from all patients before blood collection. Sampling details on geographic location and sampling period are described elsewhere [48]. The characteristics of laboratory-confirmed *P*. *falciparum* malaria at four different time points are summarized in Figure 1.

### Molecular markers

DNA was extracted from filter papers and frozen whole blood using EZNA blood DNA kit (Biofidal, Vaulx-en-Velin, France) according to the manufacturer’s recommendations. Microsatellite genotyping was performed by amplification fragment length polymorphism (AFLP) analysis. Five complex and putatively neutral microsatellite loci [32] (*Pf2689*, *7A11*, *C4M79*, *Pf2802*, and *TRAP*) were amplified by nested PCR with fluorescent end-labelled primers, as described previously [32]. Thermocycling was performed using a Biometra (Goettingen, Germany) 96-well T1 thermocycler. The accession numbers and primer sequences are summarized in Table A (Additional file 4).

Single nucleotide polymorphisms (SNPs) of *P*. *falciparum* dihydrofolate reductase (*Pfdhfr*) gene at codons 16, 51, 59, 108, and 164 associated with resistance to pyrimethamine and cycloguanil (i e, the biologically active metabolite of proguanil [51]) were determined using a primer extension method (SNaPshot®), as described in an earlier study [52]. The accession numbers are presented in Table A (Additional file 4).

AFLP and SNaPshot® products were analysed by capillary electrophoresis on polyacrylamide gels using ABI 3130XL® sequencer (Applied Biosystems®, Warrington, UK). Electrophoregram was interpreted using Genemapper® 4.0 software (Applied Biosystems®, Carlsbad, CA, USA).

### Statistical analysis

The multiplicity of plasmodial infection (MOI, defined as the number of genetically distinguishable parasites per isolate) was estimated for each isolate from the microsatellite locus that exhibited the highest number of alleles. The mean MOI for each collection year of *P*. *falciparum* population (1998, 1999, 2002, and 2009) was calculated.

The evolution of genetic diversity among Djiboutian *P*. *falciparum* populations is dependent on local transmission level and parasite flow, a source of genetic diversity. Genetic diversity was assessed by the Nei unbiased expected heterozygosity index [21], *H*_*e*_ = [*n*/(*n* − 1)][1 − *Σ*^n^_i = 1_*p*^2^_*i*_] (where *n* is the number of isolates sampled and *p*_*i*_ is the frequency of the *i*th allele) and calculated from allelic frequencies of five microsatellite loci using GENETIX software version 4.05 [53]. Pairwise comparisons of He values among the four collection periods were performed using FSTAT software version 2.9.4, with a 10,000 permutations bilateral comparison test [54].

Genetic similarity of plasmodial populations was investigated using Wright F statistic (*F*_*ST*_) [34]. Pairwise comparisons among the collection years (1998, 1999, 2002, and 2009) were performed based on microsatellite genotype frequencies using FSTAT software version 2.9.4 [35,54]. F_ST_ is a comparison of the sum of genetic variability within and between populations based on the differences in allelic frequencies. F_ST_ values were interpreted as no differentiation (0), low genetic differentiation (>0 - 0.05), moderate differentiation (0.05-0.15), and high differentiation (0.15-0.25).

Multiple correspondence analyses (MCA), also known as factorial correspondence analysis (FCA) [55] according to multilocus genotypes, were conducted to illustrate the genetic similarity of plasmodial populations during the study period. FCA was performed by considering population centroids as active points, using GENETIX software, as described in the help menu [53]. The graphical representation with 95% data concentration ellipse (i e, including 95% of the projected genotypes on the FCA plan) and centroids was obtained using R software, version R 2.15.1 [56].

The relationships between parasite genotypes were assessed using eBurst algorithm. Based on microsatellite allelic profiles, the algorithm selects the most parsimonious patterns of genotype evolution and predicts founder(s). The assignment of founders is tested by a bootstrap procedure [57]. The global optimization of the diagram based on goeBurst algorithm [58] was performed with Phyloviz software [59]. The eBurst algorithm implements a simple model of clonal expansion and diversification which is generally used to represent a population of clonal prokaryote [57]. Despite its obligatory sexual stage, *P*. *falciparum* may evolve as a clonal organism due to inbreeding [30,60-65]. A high self-fertilization rate (i.e. syngamy between genetically identical gametes) may be favoured in low malaria transmission settings [24,25,30,66]. Outbreaks are an extreme situation where the oligo-clonal spreading of parasites (i e, only few plasmodial populations propagate during epidemics) may occur [67-69]. As malaria epidemics had occurred in Djibouti with oligo-clonal expansion of plasmodial populations [32,45], eBurst diagram is particularly well adapted for the description of Djiboutian malaria situation.

Comparison between the global unstratified eBurst diagram and eBurst diagram stratified by the sampling year was performed to assess the robustness of eBurst algorithm.

The index of discriminatory power (D) was assessed in order to estimate the discriminatory power of the genotyping based on four microsatellite loci. This index is calculated from the number of genotypes and their relative frequencies. The index D is the probability that two unrelated parasites randomly sampled from studied population display different genotypes. By analogy with Nei unbiased expected heterozygosity index (He), D is an indirect measure of genotypic diversity. According to Hunter and Gaston’s formula [70], D = 1 − 1/(*N*(*N* − 1)) *Σ*^S^_j = 1_n_j_(n_j_ − 1) where *N* is the total number of parasites in the sample population, s is the total number of genotypes observed, and n_j_ is the number of strains with the jth genotype.

## Results

### Genetic diversity

When the combination of the profiles at five microsatellite loci was considered, 57, 66 and 43 genotypes were successfully obtained from 46, 61, and 32 samples collected in 1998, 1999, and 2002, respectively. Among 42 samples collected in 2009, the Pf2802 locus did not show enough profiles to perform analysis. The low success rate of Pf2802 genotyping was probably due to: i) the longer amplified fragment by the first PCR that is more sensitive to the quality of DNA template, or ii) the presence of null allele in oligo-clonal plasmodial populations. Therefore, the combination of the profiles at four microsatellite loci was considered, and 36 genotypes were observed in 2009.

Based on the microsatellite loci, a moderate genetic diversity (He) was observed during the four-year period from 1998 (He = 0.51 ± 0.20) to 2002 (He = 0.51 ± 0.14). At the end of the study period in 2009, He decreased considerably and there was no observable genetic diversity (He = 0).

The MOI showed a similar pattern, starting from the baseline mean (± SD) MOI of 1.86 ± 0.81 in 1998 to the MOI value of 1.0 in 2009. This result is particularly noteworthy, considering that the isolates were sampled in 2009 from three areas outside Djibouti city (Arta, Tadjourah, and Obock) during an interval of only a few weeks.

The numbers of alleles by locus that estimate the number of distinct genotypes were 7.8, 8.4, 6.0, and 1.0 in 1998, 1999, 2002, and 2009, respectively. These results suggested a similar tendency as MOI and He values. The results on genetic diversity, MOI, and numbers of alleles are summarized in Table 1.

**Table 1:** **Genetic diversity of *Plasmodium falciparum *and evolution of multiplicity of infection (MOI) in the Republic of Djibouti (1998–2009)**

**Populations**		**Microsatellites, individual He**					**Mean He**	**MOI**	**Mean number of alleles/ locus**
	n	7A11	C4M79	Pf 2689	Pf 2802	TRAP	[±SD]	[±SD]	
**1998**	46	0.5427(72)	0.2838(57)	0.3163(62)	0.6944(66)	0.7060(66)	0.5086[±0.20]	1.36[±0.81]	7.8
**1999**	61	0.5835(76)	0.4585(66)	0.4381(75)	0.7336(80)	0.7245(81)	0.5876[±0.14]	1.42[±0.79]	8.4
**2002**	32	0.4493(47)	0.1762(43)	0.4615(46)	0.7281(46)	0.7467(47)	0.5124[±0.23]	1.12[±0.38]	6.0
**2009**	42	0(42)	0(41)	0(42)	NA	0(36)	0	1.00	1.0

He, Nei unbiased expected heterozygosity index; *SD* standard deviation, *MOI* multiplicity of infection mean number of alleles/locus, n, number of samples, NA, no results detected. The number of genotypes successfully obtained is in parentheses.

### Parasite genetic similarity: population and individual analysis

**Figure 2:**
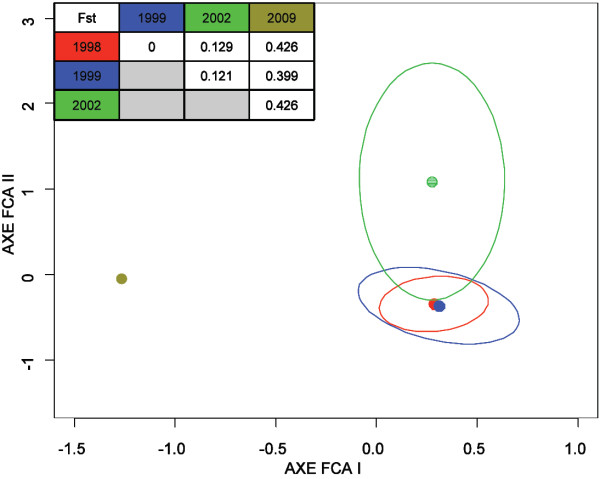
**Analysis of genetic similarity between Djiboutian *Plasmodium falciparum *populations (1998, 1999, 2002, and 2009) *Plasmodium falciparum *populations in Djibouti (1998 red dot, 1999 blue dot, 2002 light green dot, and 2009 khaki dot; sampling details are presented in the text and Figure **1**) were compared based on the genotyping of four microsatellites.** Pairwise Fst indices between the years studied are presented in the table at the top left. Statistically significant Fst values (*P* < 0.05) are underlined. Multiple correspondence analysis (also called factorial correspondence analysis) was performed with Genetix software [53], using populations’ centroid as the active point. Axes FCA I and FCA II are the first two order factors representing 51.7% and 42.4% of the total inter-centroid variance, respectively. Ellipses around the centroids enclose 95% of the projected genotypes on the FCA plan and were calculated and graphically represented using R software [56]. Genotype frequencies in 2009 did not allow ellipse representation.

The pairwise comparisons of plasmodial populations among different years of sample collection displayed statistically significant differences in Fst indices (Fst >0.12), except between 1998 and 1999 (Fst = 0). The highest Fst index (>0.39) was observed between the isolates in 2009 and those collected during the preceding years. Contiguous centroids and superimposed ellipses of plasmodial populations in 1998 and 1999 were obtained (Figure 2). Plasmodial population in 2002 appeared dissimilar from the other three populations (Fst > 0.12).

**Figure 3:**
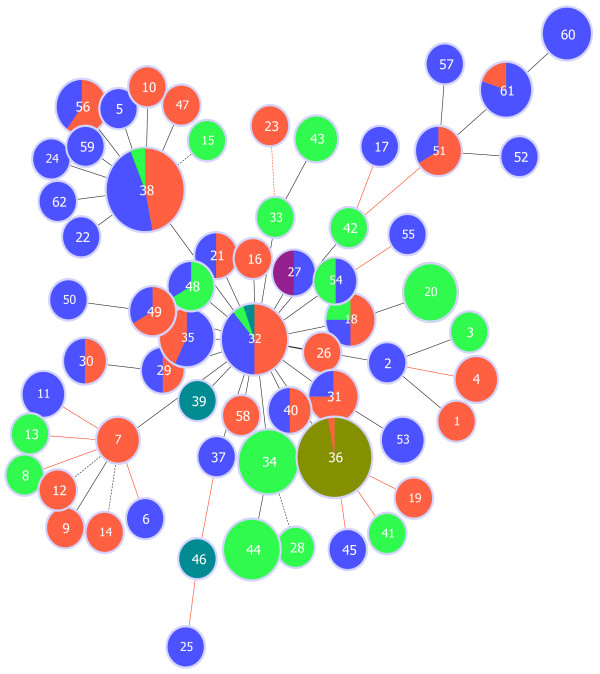
**Genetic relationship between Djiboutian *Plasmodium falciparum *genotypes observed at four time points (1998, 1999, 2002, and 2009). Unstratified analysis that does not take into account the year of sampling.** This graphical representation of relationships between closely-related genotypes of *Plasmodium falciparum* is based on eBurst algorithm [57]. A global optimization selects the more likely evolutionary pattern and highlights potential ancestral (or founding) genotype(s) of cluster of closely-related genotypes [58,59]. A total of 62 genotypes were found (numbers 1 to 62). A separation level between two distinct genotypes is represented by i) a solid line, a different profile in only one locus of microsatellite marker, i e, single-locus variant (SLV) and ii) a dotted line, a double-loci variant (DLV). The distance between genotypes in the diagram does not have any relation with genetic distance between genotypes. The area of circles depends on the number of individuals with the same genotype. The colour code is as follows: 1998 (red dot), 1999 (blue dot), 2002 (light green dot), and 2009 (khaki dot). In 2009, three malaria cases originated from Southeast Ethiopia (dark green dot) and one from Debrezeit, Ethiopia (purple dot). The proportion of each colour inside one circle depends on the number of individuals with the same genotype for the year considered. Red lines indicate relationships that are modified in stratified analysis.

**Figure 4:**
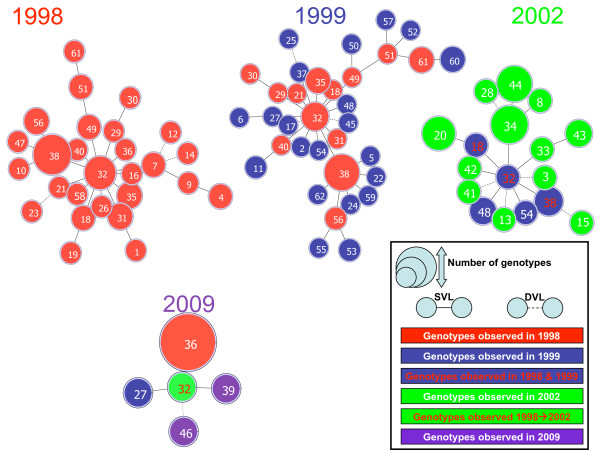
**Genetic relationship between Djiboutian *Plasmodium falciparum *genotypes observed at four time points (1998, 1999, 2002, and 2009).** Analysis stratified by sampling year. The numbers of observed genotypes were 27, 34, 17, and 5 in 1998, 1999, 2002, and 2009, respectively. The same colour code as in Figure 3 is used. Here, the colour of the text and circle corresponds to the year in which the genotypes were observed. For example, the diagram for 2002 shows new genotypes first detected in 2002 (green circle with numbers in white), genotypes previously observed in 1999 (blue circle with numbers in white), and genotypes previously observed in both 1998 and 1999 (blue circle with numbers in red). Three Ethiopian genotypes sampled in 2008 (genotypes 32, 39, and 46) were grouped with 2009 samples.

Genotype relationships provide additional information about individual relationships and yearly genotype turnover (Figure 3). Based on the analysis of four microsatellite loci, a total of 62 *P*. *falciparum* genotypes were detected in the present study. Clustering among the isolates collected during the same year was consistent with significant Fst indices between different years. During the 11-year period, the present data exhibited 10, 18, 10, and 0 private genotypes observed only in 1998, 1999, 2002 and 2009, respectively (2 genotypes observed only in 2008 were sampled in southeast Ethiopia). However, 16 genotypes were present across several study periods. Among these, 12 genotypes observed in 1998 were still present in 1999. Major genotypes were even present for three or four years. Genotype 32 was present in 1998, 1999, and 2002 and also occurred among imported malaria parasites from Ethiopia in 2008 (Figures 3 and 4). Genotype 38 also occurred in 1998, 1999, and 2002. Moreover, genotypes were closely-related from year to year. Most of the new genotypes differed at only one locus from genotypes that were observed in earlier years (i e, single-locus variant, SLV). The global, unstratified eBurst diagram, without considering the year of sampling, involved only five double-loci variants (DLVs).

In 2009, all Djiboutian isolates exhibited identical genotypes at four microsatellite loci (PF2689, C4M79, TRAP, and 7A11). This genotype, i e, genotype 36, was observed earlier in 1998. Genotype 27 observed in *P*. *falciparum* isolated from a Djiboutian military recruit who stayed in a military training camp in Debrezeit (Ethiopia) (8° 45‘ 00“ N 38° 59‘ 00“ E) for 10 months in 2009 was observed earlier in 1999. Ethiopian genotypes found in the present study were either similar to Djiboutian genotypes (genotype 32) or differed by only one locus from Djiboutian genotypes (genotypes 39 and 46).

Eburst diagram stratified by the year of detection (Figure 4) showed a similar pattern, with two major genotypes (genotypes 38 and 32) linked to numerous SLVs. This analysis avoided few links between years that had probably not occurred (13 links were modified) and pointed out more clearly the diversification of genotypes over time. Based on the eBurst diagrams of parasites collected in 1998 and 1999, six (genotypes 2, 17, 27, 37, 48, and 54) and five (genotypes 5, 22, 24, 59, and 62) new SLVs emerged in 1999 from genotypes 32 and 38, respectively. Despite this diversification, genetic lineage (56, 38, 32, 49, 51, and 61) was maintained from year to year, and major genotypes were similar (genotypes 32 and 38). To a lesser extent, in 2002, diversification occurred from an earlier major genotype (32).

Based on the frequencies of 62 genotypes observed among 225 parasites, the index of discriminatory power (D) [70] was 91%. The index D by year was 0.87, 0.89, and 0.89 for 1998, 1999, and 2002, respectively. Despite the closely-related genotypes and even recurrent genotypes observed, these genetic analyses allowed monitoring of parasite populations on a fine scale.

### Evolution of drug resistance: mutations associated with pyrimethamine resistance

Genotyping of all five *Pfdhfr* codons (16, 51, 59, 108 and 164) associated with pyrimethamine resistance was successful in 36 of 46 (78%), 50 of 61 (82%), and four of 32 (12.5%; only five samples were available after microsatellite study [32]) samples collected in 1998, 1999, and 2002, respectively. Thirteen of 42 (31%) samples obtained in 2009 were successfully genotyped, with the exception of codon 51. The prevalence of mutations in relation to the study periods is summarized in Table 2.

**Table 2:** **Evolution of the prevalence of *Pfdhfr *point mutations in codons 16, 51, 59, 108, and 164**

		**51**		**59**		**108**	
		**N (Asn) (W)**	**I (Ile) (M)**	**C (Cys) (W)**	**R (Arg) (M)**	**S (Ser) (W)**	**N (Asn) (M)**
**Year**	**N**	AAT	ATT	TGT	CGT	AGC	AAC
**1998**	36	100	0	97.2	2.8	97.2	2.8
**1999**	50	96.0	4.0	94.0	6.0	92.0	8.0
**2002**	4	100	0	100	0	100	0
**2009**	13	NA	NA	0	100	0	100

Proportions (%) of single nucleotide polymorphism are presented: *PfdhfrPlasmodium falciparum* dihydrofolate reductase, N, number of samples with at least a result at one codon; *W* wild-type, *M* mutant. NA; no results detected. Codons 16 and 164 showed only wild-type codons in all years studied.

In 1998, there was no triple mutant (defined as N51I, C59R, and S108N) and only one isolate (2.8%) carried C59R and S108N amino acid substitutions. In 1999, two (4%) isolates were triple mutants. In 2002, the prevalence of triple mutants dropped back to 0%. On the contrary, 13 (100%) isolates were double mutants (C59R and S108N) in 2009.

## Discussion

In this present study, genetic diversity was analysed and applied in innovative ways to assess the risk of malaria importation into low malaria transmission settings. Based on the results of microsatellite allele frequencies, the present study highlights an important modification in Djiboutian *P*. *falciparum* population. First, a moderate genetic diversity (He = 0.51) was observed from 1998 to 2002, with a slight increase in 1999 (He = 0.59). These results are in agreement with antigenic data and previous microsatellite analysis, which showed a similar level of genetic diversity (He = 0.53) [32]. Secondly, at the end of the study period, a significant decline in genetic diversity was recorded (He_2009_ = 0). This diminution is all the more important because, in 2009, mass screening for malaria was conducted over a period of three months in 150 sites throughout the country, and *P*. *falciparum*-positive cases were found in only three sites. These three sites are separated by hundreds of kilometres (by road), as compared with samples collected before 2009, which originated from Djibouti city only. In this context, the sampling bias in 2009 should have resulted in an opposite trend. The evolution of genetic diversity is confirmed by a similar reduction in the numbers of alleles (7.8 to 1) and the decrease in MOI at the microsatellite loci analysed during the study period.

Concerning the association between the local level of malaria transmission and plasmodial population parameters, such as genetic diversity and MOI [24,27,29,71-73], the observed significant decrease in malaria transmission in Djibouti is in agreement with the results of the recent sero-epidemiological study [48]. Interestingly, such a decrease in genetic diversity is not consistent with the level of malaria importation reported previously [32,41]. So far, even with an interruption of local malaria transmission for several months during the dry season, a long-term carriage of *P*. *falciparum*[71,74] and, more probably, imported malaria cases were able to maintain an intermediate diversity level [0.51 < He_1998-1999–2002_ < 0.58] [32]. Therefore, the absence of genetic diversity i) confirms the decline in the level of local malaria transmission, and ii) strongly suggests a reduction of imported malaria cases in Djibouti. These observations highlight the likely benefits of a coordinated malaria control programme with the neighbouring countries [75-77]. Since Ethiopia is the main source of imported malaria cases in Djibouti due to geographic proximity and socio-epidemiologic context [39,41,78], a successful Roll Back Malaria Programme in Ethiopia is expected to lower the number of imported malaria cases from Ethiopia to Djibouti.

Based on the analysis of genotype frequencies, a high Fst index (>0.15) and distant centroids (Figure 2) between sampling years strongly support a considerable plasmodial population turnover during the study period. These clusters at different time points may be attributable to subsampling of plasmodial populations from neighbouring countries (in particular Ethiopia) through human migration. As this random sampling occurred each year, the source of different strains involved in epidemics may vary over time. As fewer parasite strains are imported into Djibouti, the allelic frequencies in plasmodial populations will be less stable due to genetic drift. This phenomenon is consistent with an increase in Fst recorded from 1999 to 2009 (Fst_(1999 vs 2002)_ =0.129 and Fst_(2002 vs 2009)_ = 0.426).

Conversely, a statistically non-significant Fst and contiguous centroids between 1998 and 1999 observed during an earlier malaria situation in Djibouti are consistent with a more stable population. This particular result in 1998–1999 is probably due to i) residual malaria foci in Djibouti, leading to genetically similar populations as the source of yearly epidemics, and/or ii) higher numbers of imported malaria cases from neighbouring countries, leading to an increased genetic exchange in plasmodial populations from different countries. These observations lead to the hypothesis that Djiboutian plasmodial populations were more stable in 1998–1999. The analysis of genetic relationship showed closely related genotypes, and even similar genetic lineages, between these two years. The two most recurrent genotypes occurred nine and seven times for genotype 32 and 23 and 23 times for genotype 38 in 1998 and 1999, respectively (Figure 3 detailed in Additional file 5, Table B). To a lesser extent, similar genotypes were observed between 1999 and 2002, which suggested a common source of Djiboutian strains despite the random annual turnover due to sampling and genetic diversification (Figure 4). This data interpretation is in agreement with recurring human migratory flows across Djiboutian borders, including regular visits to Ethiopia [78] and transport of goods and persons via the international Djiboutian port [79].

The decline in genetic diversity in 2009 is due to unexpected genotype frequencies. Only one genotype was found in Djibouti in that year. In fact, after sampling 150 sites throughout the country, *P*. *falciparum* had been found in three distant sites. Taking into account both the time interval (three months) between isolate collection and distance separating the sites, identical genotypes should not be considered as the same strains but rather that they probably represent similar plasmodial populations. Moreover, genotyping analyses were performed with only five or even four microsatellite loci. Additional loci could have detected more genotypes. Nevertheless, i) the molecular system described in the present paper already attained a discriminatory index of 91% [70] and ii) as the same microsatellite markers were used throughout the 11-year study period, the present data strongly indicate a significant decrease in genetic diversity in Djibouti.

It is important to note that malaria epidemics characterized by a random clonal expansion of few plasmodial populations may lead to a rapid spread of drug resistance throughout the country. Indeed, all isolates collected in 2009 carried single nucleotide polymorphism (SNPs) associated with pyrimethamine resistance at codons 59 and 108. As the Djiboutian national anti-malarial drug policy relies on artesunate-sulphadoxine-pyrimethamine (AS-SP) combination for the first-line treatment of uncomplicated malaria, the field data presented in this paper raise the question of the necessity for a change in drug policy if molecular data are confirmed by poor clinical and parasitological response to AS-SP. However, it should be noted that i) these high percentages of *dhfr* double mutants are based on the analysis of a limited number of isolates and triple mutants have not been observed for the past 10 years in Djibouti, and ii) the observed mutants most likely reflect random fluctuations due to imported strains, as opposed to a sustained increase in the prevalence of *dhfr* mutants which would have been expected if local Djiboutian *dhfr* mutants had been selected and maintained under constant drug pressure. Molecular studies in southern Ethiopia have shown that double N51I/S108N (46%) and triple N51I/C59R/S108N *dhfr* mutants (54%) predominate in Jimma, while a large majority (>90%) of isolates are triple *dhfr* mutants in Dilla and Gambo [80-82]. Further molecular studies in Ethiopia closer to the Djiboutian border are required for the evaluation of imported malaria and spread of drug resistance in Djibouti. The results of the present study should be interpreted in the light of the prevalence of *dhfr* mutants in neighbouring countries, which may be informative to assess the risk of the spread of sulphadoxine-pyrimethamine resistance during the next epidemics in Djibouti.

Recent studies have used molecular tools to monitor plasmodial populations in regions of declining endemicity following malaria control effort or in areas of naturally low level malaria transmission ([28,69,83-86]). The most striking finding of the present study, the “clonal expansion” observed after a decline in malaria transmission, is consistent with these previous studies. Clonal expansion may occur in low transmission settings [28,61,66,69,83,87] and may be favoured by control efforts [69,84,85]. Considering the global decline in malaria prevalence over the last decade [1], more situations exhibiting epidemic or clonal expansion of parasites can be expected in the future.

The relations a between decline in malaria transmission and variations in parasite population parameters showed contradictory results, and more field data are required to establish a solid relationship between these indices and malaria situation. An expected decline in genetic diversity (He) shown in the present study (Additional file 6) was not observed in another recent study conducted in Thailand even after a considerable reduction in malaria transmission [88]. In western Kenya, a decrease in transmission has led to an increase in genetic diversity [86]. Epidemiological context and migration of human population might explain some of these discordant results. However, a comparison of results of the present study with those of other recent studies which used different molecular tools (SNP vs microsatellites) and different indices to monitor parasites should be interpreted with caution.

According to Nkhoma *et al*. [88], index based on MOI (i e, percentage of polyclonal infection) seems to be more robust or less sensible to genetic diversity resulting from migration into a study area. These authors have found a high correlation (coefficient of determination, r^2^ = 0.7967) between malaria incidence and proportion of polyclonal infections based on 96xSNP genotyping [88]. A high coefficient of determination (r^2^ = 0.99) has also been observed using SNP data [66]. The data presented in this paper (four time points, including one outbreak in 2009) did not allow a reliable estimation of correlation (r^2^ = 0.59, Additional file 7). Moreover, indices based on microsatellite loci and comparability with SNP have not been established. Nevertheless, the present data and those of other studies confirm a decrease in the percentage of polyclonal infections after a decline in malaria transmission [85].

Indices which take into account the genetic linkage disequilibrium (standardized index of association, genotype richness, and genotypic diversity) also show correlation with a decline in malaria transmission [85,88]. However, the self-fertilization rate of *P*. *falciparum* is largely unknown, which hampers the full understanding of these complex relations. Despite these limitations, analysis of relationship between closely-related genotypes in low malaria transmission settings may highlight various evolutionary patterns, such as clonal expansion, diversification, selection, migration, and residual foci, that could be modified by an intervention of malaria control programmes. Therefore, analysis of relationship between genotypes can be a powerful complementary tool to monitor *Plasmodium* populations, especially during pre-elimination and elimination phases.

## Conclusions

Molecular tools for population genetics analysis play an increasingly important role in monitoring the characteristics of *P*. *falciparum* populations. On the basis of a positive correlation between local malaria transmission, imported malaria cases and plasmodial genetic diversity, the latter was used in innovative ways to assess the risk of malaria importation into Djibouti, which is a low malaria transmission setting. The significant decrease in He, in unexpected proportions, suggests a significant decrease in local malaria transmission as well as in imported malaria. The present results indicate an evolution of Djiboutian plasmodial populations towards a malaria situation that is favourable for malaria elimination due to the presence of a few genetically distinct strains originating from the same source and spreading throughout the country. In this context, an efficient surveillance system and case management will play a major role to attain malaria elimination. Moreover, the knowledge on the geographic origin of strains would be helpful to target malaria control at the regional scale. To that end, additional molecular data from neighbouring countries, in particular from Ethiopia, are necessary. To limit additional cost required for molecular investigations, such molecular studies can use biological materials that have already been sampled (e g, RDTs for malaria and thick smear) as part of the regular activities of malaria control units in the region, provided that prior consent from patients had been obtained. Moreover, standardization of protocols (DNA extraction, genotyping and microsatellite panel) is necessary to allow direct comparison among studies and strengthen national monitoring systems in the region for a coordinated malaria control effort in the Horn of Africa.

## Abbreviations

ACT: Artemisinin-based combination therapy; AFLP: Amplification fragment length polymorphism; DLV: Double-loci variant; FCA: Factorial correspondence analysis; He: Heterozigosity expected; MCA: Multiple correspondence analysis; MOI: Multiplicity of infection; mAll: Mean number of alleles/locus; Pfdhfr: *Plasmodium falciparum* dihydrofolate reductase; SD: Standard deviation; SNP: Single nucleotide polymorphism; SLV: Single-locus variant; SP: Sulphadoxine pyrimethamine; WHO: World Health Organization.

## Competing interests

The authors declare that they have no competing interests.

## Authors‘ contributions

BAK, AA, IHF, HMA, SMB, HHG, SNA, ZA, and HYD collected the data. BAK and AP performed the genotyping analysis. BAK, HB, and MAK analysed the data. BAK wrote the first draft of the manuscript. BAK, LKB, AA, AAA, IHF, HMA, SMB, SB, SNA, HYD, BP, and CR helped to draft the manuscript. BAK, LKB, SB, BP, CR and HB participated in the interpretation of data and revised the paper. HB, BAK and CR designed the study. HB directed the research and contributed to the writing and editing of the manuscript. All authors read and approved the final manuscript.

## Supplementary Material

Additional file 1 **Classes of *P. falciparum *seroprevalence (%).** Click here for file

Additional file 2 Clusters localisation in the districts of the country. Click here for file

Additional file 3 **Numbers of malaria attacks within Djibouti city from 1998 to 2009 based on three largest Djiboutian surveillance systems: i) Peltier General Hospital (dotted squares), ii) Djiboutian National Healthcare Insurance Program (solid squares), and iii) Bouffard French Military Hospital (hatched squares) (adapted from Ollivier *et al*. 2011). Colours (red, blue, green, and purple) highlight the years with samples genotyped in the present study.** Click here for file

Additional file 4: Table A Primer sequences and amplification conditions of the 5 microsatellite loci and *Pfdhfr*: Primer sequences (5‘ → 3‘) are given for reactions no. 1 (first round) and no. 2 (second round) of the nested PCRs with fluorescent label (VIC, NED, 6-FAM or PET) and annealing temperature (Ta,°C). Thermal cycling was performed using Biometra® (Goettingen, Germany) 96-well T3 thermocycler. Size in basepairs for 3D7 reference clone. *Pfdhfr*, *P*. *falciparum* dihydrofolate reductase; Chr, chromosome. Click here for file

Additional file 5: Table B Number of distinct genotypes of *P*. *falciparum* and years based on genotyping with 4 microsatellites. MIS: Samples from Malaria Indicator Survey (described in Figure 1), D: samples from a clinical case of Djiboutian military recruit (described in Figure 1), E: South-East Ethiopian samples (described in Figure 3). Genotypes 32, 34, 36, and 38 constituted the largest number of Djiboutian isolates. Click here for file

Additional file 6 **Malaria incidence and genetic diversity in Djiboutian *P*. *falciparum *population.** The estimation of malaria incidence was based on the three largest Djiboutian surveillance systems (details in the text and Additional file 3). The calculation of genetic diversity (He) based on four microsatellite genotyping is described in material and methods section. The coefficient of determination r^2^ (r^2^ = 0. 9527;y = 0.1557ln(x) - 0.4437) suggests a positive non-linear relation between genetic diversity (He) and malaria incidence in the Republic of Djibouti. The linear coefficient of determination was lower (r^2^ = 0.5047). Click here for file

Additional file 7 **Malaria incidence and percentage of polyclonal infections in Djiboutian population of *Plasmodium falciparum *(1998, 1999, 2002, and 2009).** The estimation of malaria incidence was based on three largest Djiboutian surveillance systems (details in the text and Additional file 3). The percentage of polyclonal infections (i e, infection with multiple alleles at one or more of the four microsatellite loci) was based on the analysis of four microsatellites. Click here for file
