# Supplementary figures and images for: Population genetics analysis during the elimination process of Plasmodium falciparum in Djibouti
Source: Malar J. 2013 Jun 13;12:201. doi: 10.1186/1475-2875-12-201 (PMC3685531; doi:10.1186/1475-2875-12-201)

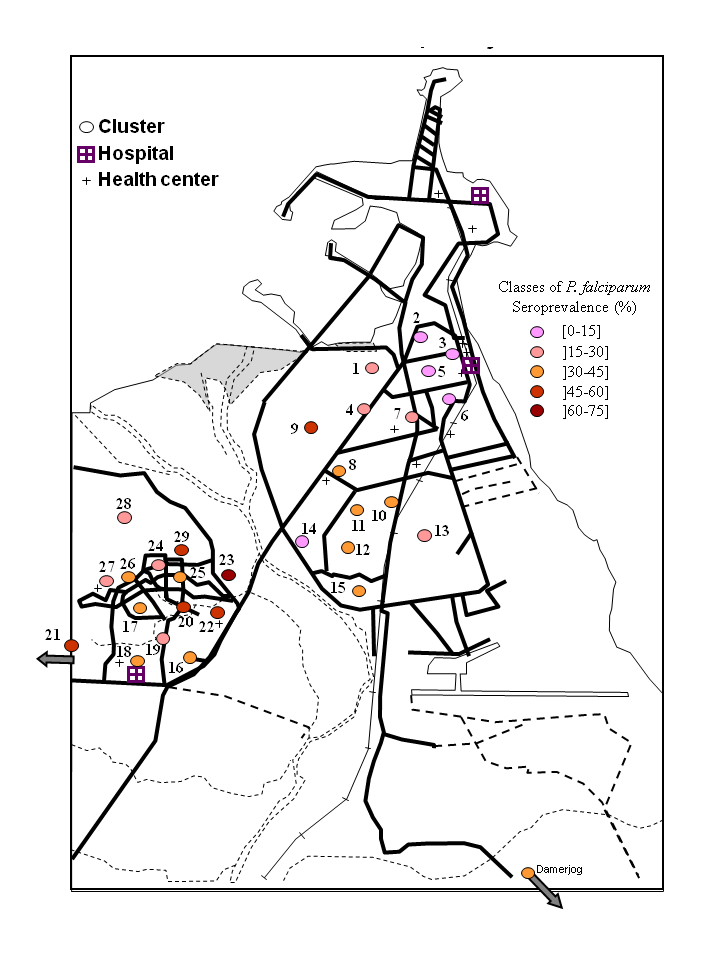

Supplement: Additional file 1 — Classes of P. falciparum seroprevalence (%). [file 1475-2875-12-201-S1.tiff]

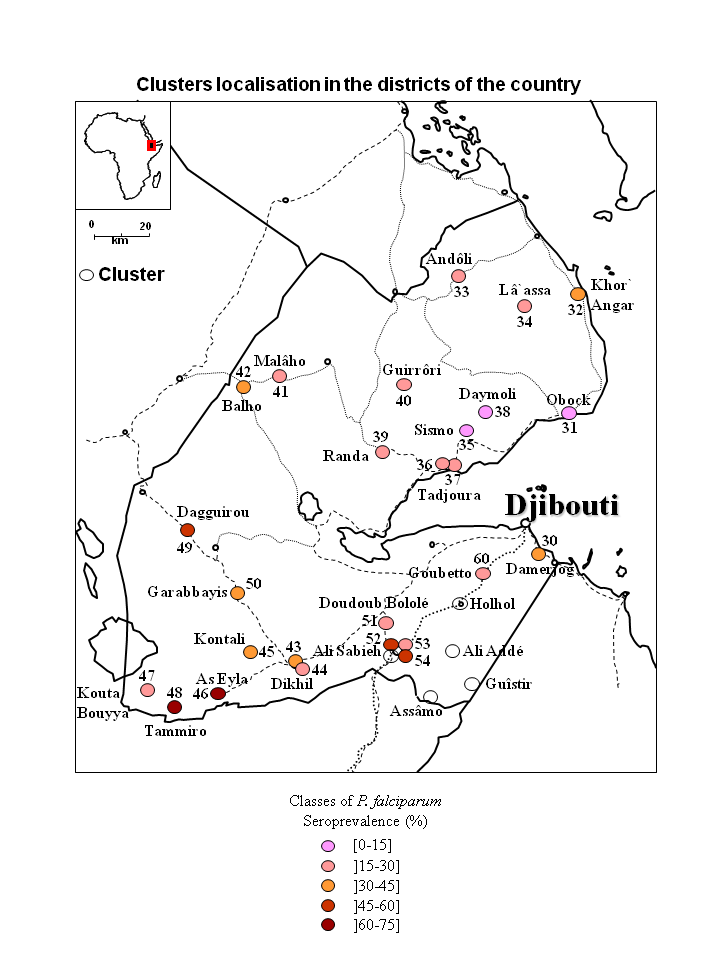

Supplement: Additional file 2 — Clusters localisation in the districts of the country. [file 1475-2875-12-201-S2.tiff]
