# Supplementary material for: Population genetics analysis during the elimination process of Plasmodium falciparum in Djibouti
Source: Malar J. 2013 Jun 13;12:201. doi: 10.1186/1475-2875-12-201 (PMC3685531; doi:10.1186/1475-2875-12-201)
Supplement: Additional file 3 — Numbers of malaria attacks within Djibouti city from 1998 to 2009 based on three largest Djiboutian surveillance systems: i) Peltier General Hospital (dotted squares), ii) Djiboutian National Healthcare Insurance Program (solid squares), and iii) Bouffard French Military Hospital (hatched squares) (adapted from Ollivier et al. 2011). Colours (red, blue, green, and purple) highlight the years with samples genotyped in the present study. [file 1475-2875-12-201-S3.doc]

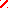

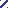

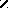

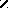

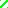

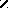

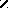

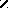

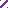

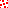

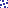

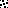

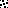

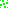

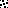

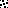

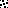

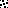

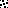

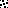

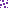

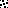

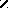


0

200

400

600

800

1000

1200

1400

1600

1998

1999

2000

2001

2002

2003

2004

2005

2006

2007

2008

2009

years

Peltier General hospital

Bouffard French Military Hospital

Djiboutian National Healthcare Insurance Program

Numbers of malaria attacks

Additional file 3: Numbers of malaria attacks within Djibouti city from 1998 to 2009 based on three largest Djiboutian surveillance systems: i) Peltier General Hospital (dotted squares), ii) Djiboutian National Healthcare Insurance Program (solid squares), and iii) Bouffard French Military Hospital (hatched squares) (adapted from Ollivier *et al* 2011). Colours (red, blue, green, and purple) highlight the years with samples genotyped in the present study.
