# Supplementary material for: Population genetics analysis during the elimination process of Plasmodium falciparum in Djibouti
Source: Malar J. 2013 Jun 13;12:201. doi: 10.1186/1475-2875-12-201 (PMC3685531; doi:10.1186/1475-2875-12-201)
Supplement: Additional file 4: Table A — Primer sequences and amplification conditions of the 5 microsatellite loci and Pfdhfr: Primer sequences (5‘ → 3‘) are given for reactions no. 1 (first round) and no. 2 (second round) of the nested PCRs with fluorescent label (VIC, NED, 6-FAM or PET) and annealing temperature (Ta,°C). Thermal cycling was performed using Biometra® (Goettingen, Germany) 96-well T3 thermocycler. Size in basepairs for 3D7 reference clone. Pfdhfr, P. falciparum dihydrofolate reductase; Chr, chromosome. [file 1475-2875-12-201-S4.doc]

| **Loci** | **GenBank accession no.** | **Size, bp** | **Chr** | | **Primer ID** | **Primer sequences for 1^st^ and 2^nd^ rounds of nested PCR (5′ → 3′)** | | **Ta, °C** |
| --- | --- | --- | --- | --- | --- | --- | --- | --- |
| Pf2689 | [G37854](http://www.ncbi.nlm.nih.gov/nuccore/3015460) | 86 | 2 | Pf2689 PCR1 | | TTA ACC TTA TAG CTT CAG AG | | 56 |
|  |  |  |  |  | | TCT TCT TCA CTT ACA TTA AAG | |  |
|  |  |  |  | Pf2689 PCR2 | | TAT GCA CAC ACG TTT CTA | | 54 |
|  |  |  |  |  | | 6-FAM—CTC CAA GGC ATT CAC GTA | |  |
| 7A11 | [G38831](http://www.ncbi.nlm.nih.gov/nuccore/3319145) | 92 | 7 | 7A11 PCR1 | | ACA TAT TAT TTC TTC GTA A | | 53 |
|  |  |  |  |  | | TTA TCT CTT CTC TGA GTA A | |  |
|  |  |  |  | 7A11 PCR2 | | ATG TGT AAG GAG ATA GTA TA | | 54 |
|  |  |  |  |  | | 6-FAM—CAA CTT TCT CTT TTT AAA TAT TAC | |  |
| C4M79 | [G42726](http://www.ncbi.nlm.nih.gov/nuccore/4337376) | 220 | 3 | C4M79 PCR1 | | TTT TGT AGG AAC ATG TAA | | 53 |
|  |  |  |  |  | | GGA GAC TAG CTC TAC AAT A | |  |
|  |  |  |  | C4M79 PCR2 | | TTT ATA TCA AGA ATG ACA ACC | | 57 |
|  |  |  |  |  | | NED—TAG CAA CAA TAA ACA ATA TGG | |  |
| Pf2802 | [G37818](http://www.ncbi.nlm.nih.gov/nuccore/3015424) | 136 | 5 | Pf2802 PCR1 | | GAT GCT TAG TTT AAT CTT ATA ACA AAT A | | 60 |
|  |  |  |  |  | | GAC TTA CTT TCT TAC ATA AAA TCA TTA AC | |  |
|  |  |  |  | Pf2802 PCR2 | | GTA TAA AAG GAA ATA CCT A | | 52 |
|  |  |  |  |  | | NED—CAG ACT ATC TTA AGG GAA | |  |
| TRAP | [G37858](http://www.ncbi.nlm.nih.gov/nuccore/3015464) | 134 | 3 | TRAP PCR1 | | ATA AAA CAA ATT ACC GAG TA | | 56 |
|  |  |  |  |  | | ACA ATT CAG ATT ACC TGA A | |  |
|  |  |  |  | TRAP PCR2 | | CAT AAT AGT AGC AAG AGA | | 49 |
|  |  |  |  |  | | PET—GAT TAT ATA TAG CGA TTT AC | |  |
| Pfdhfr |  |  | 4 | Pfdhfr primers (template) | | | |  |
|  |  |  |  | dhfr PCR1 | | | TTC TCC TTT TTA TGA TGG AAC AAG T | 56 |
|  |  |  |  |  | | | ATA TTT GAA AAT CAT TTG GAT GTA TAG |  |
|  |  |  |  | dhfr PCR2 | | | ACG TTT TCG ATA TTT ATG C | 47 |
|  |  |  |  |  | | | TCA CAT TCA TAT GTA CTA TTT ATT C |  |
|  |  |  |  | Pfdhfr primers (SNaPshot) | | | |  |
|  |  |  |  | dhfr51-f | | | AGG AGT ATT ACC ATG GAA ATG TA |  |
|  |  |  |  | dhfr16-r | | | gactgactCTC ATT TTT GCT TTC A AC CTT ACA ACA T |  |
|  |  |  |  | dhfr108-f | | | gactgactACA AAA TGT TGT AGT TAT GGG AAG AAC AA |  |
|  |  |  |  | dhfr164-r | | | ctgactgactgactgactAAT TCT TGA TAA ACA ACG GAA CCT CCT A |  |
|  |  |  |  | dhfr59-r | | | ctgactgactgactgactgactTGA TTC ATT CAC ATA TGT TGT AAC TGC AC |  |
|  |  |  |  |  | | |  |  |
|  |  |  |  |  | | |  |  |

**Additional file 4 – Table A: Primer sequences and amplification conditions of the 5 microsatellite loci and** **Pfdhfr:** Primer sequences (5′ → 3′) are given for reactions no. 1 (first round) and no. 2 (second round) of the nested PCRs with fluorescent label (VIC, NED, 6-FAM or PET) and annealing temperature (Ta, °C). Thermal cycling was performed using Biometra® (Goettingen, Germany) 96-well T3 thermocycler. Size in basepairs for 3D7 reference clone. *Pfdhfr*, *P. falciparum* dihydrofolate reductase; Chr, chromosome.
