# Supplementary material for: Population genetics analysis during the elimination process of Plasmodium falciparum in Djibouti
Source: Malar J. 2013 Jun 13;12:201. doi: 10.1186/1475-2875-12-201 (PMC3685531; doi:10.1186/1475-2875-12-201)
Supplement: Additional file 5: Table B — Number of distinct genotypes of P. falciparum and years based on genotyping with 4 microsatellites. MIS: Samples from Malaria Indicator Survey (described in Figure 1), D: samples from a clinical case of Djiboutian military recruit (described in Figure 1), E: South-East Ethiopian samples (described in Figure 3). Genotypes 32, 34, 36, and 38 constituted the largest number of Djiboutian isolates. [file 1475-2875-12-201-S5.doc]

| **Genotype number** | **Year** | | | | | | **Total** |
| --- | --- | --- | --- | --- | --- | --- | --- |
|  | **1998** | **1999** | **2002** | **2009 MIS** | **2009 D** | **2009 E** |  |
| **1** | 1 |  |  |  |  |  | **1** |
| **2** |  | 1 |  |  |  |  | **1** |
| **3** |  |  | 1 |  |  |  | **1** |
| **4** | 2 |  |  |  |  |  | **2** |
| **5** |  | 1 |  |  |  |  | **1** |
| **6** |  | 1 |  |  |  |  | **1** |
| **7** | 2 |  |  |  |  |  | **2** |
| **8** |  |  | 1 |  |  |  | **1** |
| **9** | 1 |  |  |  |  |  | **1** |
| **10** | 1 |  |  |  |  |  | **1** |
| **11** |  | 2 |  |  |  |  | **2** |
| **12** | 1 |  |  |  |  |  | **1** |
| **13** |  |  | 1 |  |  |  | **1** |
| **14** | 1 |  |  |  |  |  | **1** |
| **15** |  |  | 1 |  |  |  | **1** |
| **16** | 1 |  |  |  |  |  | **1** |
| **17** |  | 1 |  |  |  |  | **1** |
| **18** | 2 | 1 | 1 |  |  |  | **4** |
| **19** | 1 |  |  |  |  |  | **1** |
| **20** |  |  | **6** |  |  |  | **6** |
| **21** | 1 | 1 |  |  |  |  | **2** |
| **22** |  | 1 |  |  |  |  | **1** |
| **23** | 1 |  |  |  |  |  | **1** |
| **24** |  | 1 |  |  |  |  | **1** |
| **25** |  | 1 |  |  |  |  | **1** |
| **26** | 1 |  |  |  |  |  | **1** |
| **27** |  | 1 |  |  | 1 |  | **2** |
| **28** |  |  | 1 |  |  |  | **1** |
| **29** | 1 | 1 |  |  |  |  | **2** |
| **30** | 1 | 1 |  |  |  |  | **2** |
| **31** | 3 | 1 |  |  |  |  | **4** |
| **32** | **9** | **7** | 1 |  |  | 1 | **18** |
| **33** |  |  | 1 |  |  |  | **1** |
| **34** |  |  | 11 |  |  |  | **11** |
| **35** | 3 | 4 |  |  |  |  | **7** |
| **36** | 1 |  |  | **36** |  |  | **37** |
| **37** |  | 1 |  |  |  |  | **1** |
| **38** | **23** | **23** | 3 |  |  |  | **49** |
| **39** |  |  |  |  |  | 1 | **1** |
| **40** | 1 | 1 |  |  |  |  | **2** |
| **41** |  |  | 1 |  |  |  | **1** |
| **42** |  |  | 1 |  |  |  | **1** |
| **43** |  |  | 2 |  |  |  | **2** |
| **44** |  |  | **8** |  |  |  | **8** |
| **45** |  | 1 |  |  |  |  | **1** |
| **46** |  |  |  |  |  | 1 | **1** |
| **47** | 1 |  |  |  |  |  | **1** |
| **48** |  | 1 | 2 |  |  |  | **3** |
| **49** | 2 | 1 |  |  |  |  | **3** |
| **50** |  | 1 |  |  |  |  | **1** |
| **51** | 2 | 1 |  |  |  |  | **3** |
| **52** |  | 1 |  |  |  |  | **1** |
| **53** |  | 2 |  |  |  |  | **2** |
| **54** |  | 1 | 1 |  |  |  | **2** |
| **55** |  | 1 |  |  |  |  | **1** |
| **56** | 3 | 2 |  |  |  |  | **5** |
| **57** |  | 1 |  |  |  |  | **1** |
| **58** | 1 |  |  |  |  |  | **1** |
| **59** |  | 1 |  |  |  |  | **1** |
| **60** |  | 4 |  |  |  |  | **4** |
| **61** | 1 | 4 |  |  |  |  | **5** |
| **62** |  | 1 |  |  |  |  | **1** |
| **Total** | **68** | **74** | **43** | **36** | **1** | **3** | **225** |

### Additional file 5 – Table B: Number of distinct genotypes of *P. falciparum* and years based on genotyping with 4 microsatellites. MIS: Samples from Malaria Indicator Survey (described in Figure 1), D: samples from a clinical case of Djiboutian military recruit (described in Figure 1), E: South-East Ethiopian samples (described in Figure 3). Genotypes 32, 34, 36, and 38 constituted the largest number of Djiboutian isolates.
