# Supplementary material for: Population genetics analysis during the elimination process of Plasmodium falciparum in Djibouti
Source: Malar J. 2013 Jun 13;12:201. doi: 10.1186/1475-2875-12-201 (PMC3685531; doi:10.1186/1475-2875-12-201)
Supplement: Additional file 6 — Malaria incidence and genetic diversity in Djiboutian P. falciparum population. The estimation of malaria incidence was based on the three largest Djiboutian surveillance systems (details in the text and Additional file 3). The calculation of genetic diversity (He) based on four microsatellite genotyping is described in material and methods section. The coefficient of determination r2 (r2 = 0. 9527;y = 0.1557ln(x) - 0.4437) suggests a positive non-linear relation between genetic diversity (He) and malaria incidence in the Republic of Djibouti. The linear coefficient of determination was lower (r2 = 0.5047). [file 1475-2875-12-201-S6.doc]

Malaria incidence and genetic diversity in Djiboutian *P. falciparum* population (1998, 1999, 2002, and 2009)

Genetic diversity He

Annual number of malaria attacks

**Additional file 6: malaria incidence and genetic diversity in Djiboutian *P. falciparum* population.** The estimation of malaria incidence was based on the three largest Djiboutian surveillance systems (details in the text and Additional file 3). The calculation of genetic diversity (He) based on four microsatellite genotyping is described in material and methods section. The coefficient of determination r² (r² = 0. 9527;y = 0.1557ln(x) - 0.4437) suggests a positive non-linear relation between genetic diversity (He) and malaria incidence in the Republic of Djibouti. The linear coefficient of determination was lower (r²=0.5047).
