# Supplementary material for: Population genetics analysis during the elimination process of Plasmodium falciparum in Djibouti
Source: Malar J. 2013 Jun 13;12:201. doi: 10.1186/1475-2875-12-201 (PMC3685531; doi:10.1186/1475-2875-12-201)
Supplement: Additional file 7 — Malaria incidence and percentage of polyclonal infections in Djiboutian population of Plasmodium falciparum (1998, 1999, 2002, and 2009). The estimation of malaria incidence was based on three largest Djiboutian surveillance systems (details in the text and Additional file 3). The percentage of polyclonal infections (i e, infection with multiple alleles at one or more of the four microsatellite loci) was based on the analysis of four microsatellites. [file 1475-2875-12-201-S7.doc]

**Malaria incidence and percentage of polyclonal infections in Djiboutian population of *Plasmodium falciparum* (1998, 1999, 2002, and 2009)**

Percentage of poly-clonal infections (%)

1998

1999

2002

2009

R² = 0.5469

0

5

10

15

20

25

30

35

0

200

400

600

800

1000

1200

1400

Annual number of malaria attacks

**Additional file 7: Malaria incidence and percentage of polyclonal infections in Djiboutian population of *Plasmodium falciparum* (1998, 1999, 2002, and 2009)**. The estimation of malaria incidence was based on three largest Djiboutian surveillance systems (details in the text and Additional file 3). The percentage of polyclonal infections (i e, infection with multiple alleles at one or more of the four microsatellite loci) was based on the analysis of four microsatellites.
